# A Dichotomous Analysis of Diseases Founded on Their Symptoms

**Published:** 1873-06

**Authors:** W. T. Grant

**Affiliations:** Georgia


### A DICHOTOMOUS ANALYSIS OF DISEASES FOUNDED ON THEIR SYMPTOMS.

BY W. T. GRANT, M.D., OF GEORGIA.

In offering to the profession the following analysis, I wish to say a few words. It may appear to be a very easy thing to construct such analyses, but on trial it will be far from easy. The first idea that would probably present itself to any one beginning such a work would be to divide all symptoms into general and special, or constitutional and local symptoms, and then use them in the various dichotomes. But it will very quickly be found that the general or constitutional symptoms are almost worthless and unavailable for the purpose, from the fact that they are common to so many diseases, and are rarely sufficiently defiinite to be used as characteristics. The consequence is, that the special symptoms will be our only resource, except in the few first very general divisions of the subject. And every physician knows how few reliable diagnostic or pathognomonic symptoms there are even among the special symptoms of disease. Our diagnoses depend more upon the symptoms as they are *grouped* in the different diseases, than upon any distinct or individual symptoms. Indeed, very few diseases furnish each a single characteristic symptom.

I can see no good reason why syphilis and gonorrhœa are classed as surgical, rather than as medical diseases, and I have, therefore, classified one of them with the other diseases which the physician is called on to treat. I should like to know if there is any other good reason than custom for classing them as surgical diseases. I think that their affinities would remove them from the province of surgery, and, if custom is the only reason for keeping them there, it would be an improvement in our nosology to remove them to that position which their analogies point out as most proper for them.

The dichotomous analysis may be applied to surgery, and also to obstetric diseases. And I have thought somewhat of similar analyses in connection with materia medica, to be based upon the physiological effects of remedies. But I have no facilities at present for such work, as the peripatetic character of my engagements utterly precludes any possibility of my having books, and a reference to many works on materia medica would be essential in the construction of such an analysis. My own experience, one work on practice, and about half a dozen medical journals, are the data on which I have had to depend in arranging the following analysis of diseases.

Another and far more difficult application of this principle is to medical botany. But I will say nothing more on that subject, as I propose to give an entire monograph to it—perhaps two. I believe this to be something new under the sun, but it is a vast subject, and probably the devotion of a lifetime to it would not do more than introduce the investigator upon its threshold. But of that in due time.

A correct diagnosis is so very important in the practice of medicine, that anything which tends to simplify it must be meritorious. And as the following analysis is what may be called a *tabulated*

diagnosis, or diagnosis reduced to its utmost simplicity, it is for the profession to decide whether there is any merit in the work.

There is perhaps some need for an explanation of this analysis. I therefore give the following:

We have a case of disease before us which we wish to diagnose; we refer to the first division of the analysis, and ask, Is this disease attended with an eruption on the skin or not? (See the first division in the analysis, marked No. 1, in the left column.) Our case has an eruption on the skin. The figure in the right column opposite this is 43. This is a reference figure, and refers us to No. 43 in the left column, which column is numbered regularly from 1 up to 55. We look down the left-hand column, therefore, for 43. Here we have another division; is *high* fever an initial symptom or not of the disease? It is not, we find, by referring to the case. Figure 47 stands opposite this statement, and we look on down the left column for 47. Here occurs another question, Is the disease clearly eruptive, or is it simply discolored? We know by the case that is clearly eruptive. So our next reference on the right is 49. Looking on down as before, when we find 49 we see that we have here several questions: Is our disease papulous? or vesicular? or pustulous? or squamous? or tubercular? or bullar? We decide which again by reference to the case before us. Suppose we find it vesicular, our reference figure on the right is then 51. On looking down for 51, we find three divisions, and that the first corresponds best with the case we are studying—that is, the case has an eruption "occurring in circumscribed patches, upon an inflamed base, which extends somewhat beyond their margins." Here we find the name of our disease, which is herpes.

In the same manner we can trace out the name of any other disease, and if our analysis be exhaustive and correct in every particular, we may rely upon the result. I do not claim that the following analysis is by any means perfect, but I believe that it is correct so far as it goes. It is out of my power now to make it perfect, and those who wish a better may construct it for themselves:

1. Disease attended with an eruption on skin............. 43
1. Disease not attended with an eruption on skin......... 2
2. Disease attended with an unusual discharge from some of the orifices of the body; or the natural discharge altered, 3

2. Disease not attended with an unusual discharge from some of the orifices of the body; and the natural discharge not altered.......................................... 29
3. The discharge by the mouth.......................... 4
3. The discharge by the nose........................... 17
3. The discharge by the anus........................... 19
3. The discharge by the urethra........................ 22
4. Discharge produced by cough........................ 5
4. Discharge not produced by cough.................... 8
5. Discharge bloody, or containing blood................ 6
5. Discharge not bloody, or not containing blood......... 7
6. Discharge extremely viscid and tenaceous; pain in chest acute; percussion dull; a blush on left cheek.....*Pneumonia.*
6. Discharge not extremely viscid and tenaceous; pain rather a feeling of soreness; percussion clear......*Acute Bronchitis.*
6. Discharge purulent and offensive; breath fetid. *Gangrene of Lung.*
7. Pain increased by full inspiration, or by pressure on intercostal spaces..................................*Pleurisy.*
7. Pain not increased by full inspiration, and not by pressure on intercostal spaces..............................*Phthisis.*
8. Vomiting excessive and persistent, and discharge containing fœcal matter.............................*Intussusception.*
8. Discharge containing blood.......................... 9
8. Discharge not containing blood...................... 10
9. Discharged blood coagulated and dark, and mixed with contents of stomach; no blood in the nose......*Hœmatemesis.*
9. Discharged blood bright red, frothy, unmixed; with other chest symptoms...............................*Hœmoptysis.*
10. Disease decidedly febrile from first (*i. e.*, the fever a prominent symptom).................................... 11
10. Disease not decidedly febrile from first (*i. e.*, any fever present is an accident to the disease.................. 13
11. Fever decidedly remitting.......................... 12
11. Fever not decidedly remitting, or is continued; forehead dark dusky colored..........................*Yellow Fever.*
12. Surface cold; heat circumscribed on one part of body; thirst and internal heat intense; pulse thready, and heart laboring....................................*Congestive Chill.*

12. Surface not cold; heat not circumscribed on any part of body; thirst and internal heat not intense; pulse not thready, and heart not laboring............. *Remittent Fever.*
12. Pulse bounding; pupils contracted; painful sensitiveness to light; pain in head continuous; great muscular pain.. *Meningitis.*
13. Seat of greatest distress in head or throat............. 14
13. Seat of greatest distress in abdomen.................. 16
14. Disease in throat or mouth......................... 15
14. Disease in head; little fever; head pain paroxysmal; pulse irregular; sudden flushes of face; feeling of numbness or of being asleep in different parts; no undue sensitiveness to light.................................. *Cerebritis.*
15. Heat and uneasiness in fauces; swallowing difficult; tonsil swollen; pain sometimes shooting into ear...... *Tonsilitis.*
15. Heat and pain referred to lower end of pharynx, or cordiac extremity of stomach.................... *Œsophagitis.*
15. Gums inflamed with excessive discharge of saliva without effort.................................... *Ptyalism.*
16. Pain lancinating; countenance cachectic, *i. e.*, a peculiar yellowish white waxen appearance; an epigastric tumor discoverable......................... *Cancer of Stomach.*
16. Abdomen retracted, with a knotted feeling of abdominal muscles; constipation obstinate, with the feeling that an operation would relieve; a pale bluish gray line along the margins of the gums; cramps in extremities; pain paroxysmal, with incomplete remissions........ *Colica Pictonum.*
16. Belching common; a sense of uneasiness not amounting to pain, greatest when the stomach is empty; a consciousness that the food is not properly digested; hot and stimulating drinks afford a temporary relief.......... *Dyspepsia.*
16. Pressure on abdomen very painful; abdomen tumefied; pain sharp, severe, but not paroxysmal; face expresses great anxiety and distress; tendency to constipation great, *Peritonitis.*
16. Pain under right clevicle or in right shoulder (or sometimes in left), *together with* tenderness in *right* hypochondrium, which is commonly tumefied; cough and dyspnœa often present................................ *Hepatitis.*

16. Deep-seated pain, with swelling in *left* hypochondrium; pain increased by pressure; generally present with intermittent fever; if idiopathic, symptoms of other diseases absent................ .....................*Splenitis.*
16. Deep-seated pain in region of kidney, shooting down into groin or thigh; urine scanty, frequently discharged; with retraction of testicles............................*Nephritis.*
16. Pain similar to nephritis, but the urine is highly albuminous, and frothy when discharged or shaken; generally attended with dropsy..........................*Bright's Disease.*
17. Discharge consisting of simple blood.............*Epistaxis.*
17. Discharge not containing blood...................... 18
18. Disease preceded by sneezing; discharge a thin, transparent watery fluid, sometimes quite acrid; eyes often red and watery..................................*Coryza.*
18. A feeling of stiffness and uneasiness in the nostril; efforts sometimes made to clear out the nostrils by forcible inspispiration; discharge purulent, often offensive; a scab, detached from an ulcer, is sometimes thrown out........*Ozena.*
19. Discharge bloody................................ 21
19. Discharge not bloody......... ................... 20
20. Alvine discharges watery, and simply increased in frequency and quantity........... ...............*Diarrhœa.*
20. Vomiting simultaneous with the diarrhœa, attended with pain in the bowels, *and always consequent on some imprudence in eating*......... ..............*Cholera Morbus.*
20. Vomiting and diarrhœa simultaneous and excessive, *attended with violent cramps in the extremities;* arising from an epidemic influence...................*Epidemic Cholera.*
20. *A summer disease of children,* with vomiting and purging; abdomen usually flat and sunken; child usually sleeps throughout the disease, with the eyes half open........ *Cholera Infantum.*
21. Alvine discharges fluid and bloody, or attended with tenesmus, *i. e.,* a constant desire but inability to discharge anything; colicky, griping pains in bowels........*Dysentery.*
21. *Shooting pains,* arising from a particular spot in abdomen, preceded for a long time by an uneasy feeling there, and followed by a tumor; if the upper bowel is affected, there will be vomiting—if the lower, there is glairy, mucous or sanious, and often very offensive discharges from the bowels ....... ........................ *Cancer of Bowels.*

21. A tumor on the verge of the anus, either just within or just without, often alternately enlarging and contracting, attended with more or less pain and uneasiness; if inflamed, there is bleeding, with considerable pain and tenesmus ................................ *Hæmorrhoids.*

21. Simple discharge of dark-colored blood, without symptoms of any other disease ...................... *Melæna.*

22. Urine affected in quantity ........................ 23

22. Urine not affected in quantity ............. ......... 26

23. Quantity greater than usual ....................... 24

23. Quantity less than usual .......................... 25

24. Simple increase of urine, without other symptoms; urine pale or light yellow; specific gravity low; no sugar nor albumen in it ................................ *Diuresis.*

24. Gradual emaciation with dyspeptic symptoms; urine sweet; also contains albumen, as shown by the combined effects of heat and nitric acid; chilliness common; thirst and appetite often great ............................ *Diabetes.*

25. Little or no discharge of urine, followed by hiccough, nausea and vomiting, with dullness approximating to coma, with no symptoms of other disease; breath or sweat with a urinous odor ................. *Suppression of Urine.*

25. Retention seldom complete; pain greater than in suppression, and increased by pressure on loin; being a sequela, it is always preceded by some other disease—*i. e.*, gonorrhœa, &c ......................... *Retention of Urine.*

26. Urine the only discharge .......................... 27

26. Other discharge beside urine ...................... 28

27. Pain above and behind the pubes, much increased by pressure, with constant desire but ineffectual efforts to empty the bladder ............................ *Cystitis.*

27. Simple involuntary escape of urine, without symptoms of other disease ..................... *Incontinence of Urine.*

28. Discharge yellowish or cream color and thickened; tenderness of some part of urethra; pain great on urinating or erection; always following close upon sexual intercourse.......................................*Gonorrhœa.*

28. A simple whitish discharge, without other symptoms, and generally following a strain; no pain while urinating, &c..........................................*Gleet.*

28. Urine becoming turbid or muddy, and depositing a sediment........................................*Lithiasis.*

28. Involuntary seminal emissions, with incomplete or no erection, occurring day or night, unattended with pain, and often simply dribbling away...........*Spermatorrhœa.*

29. Fever a regular and leading symptom, always present... 30

29. Fever not a regular and leading symptom (an accident of the disease if present)................................ 32

30. Fever paroxysmal, and occurring with regular intervals, (daily, &c.)............ ................*Intermittent Fever.*

30. Fever not paroxysmal but continued; occasionally with slight remissions................................ 31

31. Attack slow and insidious; abdomen tympanitic, with borborygmi, and an eruption which often extends to the breast, and is affected by pressure; prostration progressive; pain in head confined to one spot and persistent; bowels inclined to be free; disease always sporadic, and rarely attacking an elderly person...........*Typhoid Fever.*

31. Generally more abrupt in commencement; eruption easily affected by pressure, and may cover the entire person; a peculiar odor about patient; bowels costive; abdomen flat and not usually tympanitic; contagious; rarely sporadic, and often attacking elderly people............*Typhus Fever.*

32. Main seat of the disease in the head or throat.......... 33

32. Main seat of the disease in the chest................. 34

32. Main seat of the disease in the abdomen.............. 35

32. Main seat of the disease in the extremities............ 36

32. Disease not local, but constitutional, but may exhibit itself locally sometimes.................................. 37

33. Inability to swallow, with pain in pharynx increased by pressure...................................*Pharyngitis.*

33. Cough greatly prolonged, the intervening inspiration attended with loud sound, and the cough generally ends by vomiting.......................................*Pertussis.*

33. Commonly confined to infants; gradual enlargement of head; satures open and exhibit fluctuation; expression of face usually stupid; bowels costive; special senses dull.. *Hydrocephalus.*

33. Pain, heat and swelling just under the ear, with more or less inability to open the mouth; metastasis common. *Parotitis.*

34. More or less dyspnœa, always increased by pressure on lungs; chest cylindrical; the hollows above and below clavicles filled up; intercostal spaces also full; percussion gives a very clear and cavernous sound; auscultation shows the respiratory murmur to be feeble..... *Emphysema of Lung.*

34. Dyspnœa paroxysmal, with greater or less intervals, during which patient enjoys good health, if they be long; respiration attended with a peculiar wheezing noise ...... *Asthma.*

34. Attack sudden; abdomen swollen and at length much distended; *pressure gives ease;* symptoms of other disease absent.................................. *Flatulent Colic.*

34. Attack sudden; abdomen contracted, the bowels sometimes knotted; pain paroxysmal and tearing, and *not relieved* by pressure; bowels constipated.................. *Cramp Colic.*

34. Attack not always sudden, with bilious vomiting; *a paroxysm of pain always follows any movement of the patient.* *Bilious Colic.*

35. Occurs mostly in children; more or less pain in bowels; *picking or scratching at the nose or anus;* diarrhœa or constipation, either may be present; abdomen generally rounded; appetite capricious; sometimes convulsions, and even strabismus; irritability and wakefulness great... *Worms.*

35. Entire abdomen swollen, exhibiting fluctuation; pain absent; percussion gives a flat and dull sound; the fluid gravitates according to position; in the supine position, the front is flat and the sides bulge.................. *Ascites.*

36. Acute pain in a *small* joint, *almost always in the great toe,* and violently intense; the attack generally beginning in the latter part of the night; otherwise the health is vigorous; is a disease of paroxysms; *superficial veins of affected part turgescent,* which part becomes œdematous, followed by desquamation; attendant fever remittent..... *Gout.*

36. An unaccountable tumor, compressible, with pulsation synchronous with heart's action, and having a rasping, bellows sound; pressure above diminishes, and below swells it.................................. *Aneurism.*
36. Pain in groin and thigh, followed by great swelling of thigh, which is unnaturally white, shining, hot and elastic; not pitting on pressure; femoral vein can usually be felt as a hard, knotted cord; usually attacks females soon after labor, and has constitutional symptoms.... *Phlegmasia Dolens.*
36. *Larger joints* affected, generally the knee or ankle first; pain aggravated by bad weather; pain metastatic and followed by swelling of affected joint, which do not subside with the pain; at first fugitive pains, dull and aching, constitute the disease........................ *Rheumatism.*
37. Disease attended with great pain and suffering......... 38
37. Disease not attended with great pain and suffering...... 40
38. Disease attended with spasms....................... 39
38. Disease not attended with spasms; pain *intense, lancinating, darting, always following* the course of some nerve, as the fifth pair, the sciatic, or some other, and consisting of a succession of violent twinges of pain........ *Neuralgia.*
39. Disease preceded by the bite of some rabid animal, and the first distress occurs in the cicatrix, soon followed by spasms in the fauces on swallowing anything, especially fluids; the very appearance of liquids at length excites spasms; patient fully rational................ *Hydrophobia.*
39. Stiffness in back of neck and rigidity of pains; darting pains in epigastrium; transient spasms of voluntary muscles, attended with pain; at length permanent spasm or rigidity of some set of voluntary muscles takes place, flying abruptly from one set of muscles to another... *Tetanus.*
40. Disease one of spasms or convulsions.................. 41
40. Disease not one of spasms or convulsions............ 42
41. Attack sudden; patient unconscious; convulsions attended with *foaming at mouth,* and ending in a comatose sleep. *Epilepsy.*
41. Attack gradual; the voluntary muscles are called into action by the will, but do not obey it; there is no spasm, no pain, no fever and no coma; the voluntary muscles seem only to have gone crazy...................... *Chorea.*

42. Disease sudden or gradual; often preceded by more or less impairment of the senses and motion; patient usually falls down unconscious, without sensation or motion; face without expression and suffused; *pulse full, strong, slow, and often intermits;* respiration commonly stertorous. *Apoplexy.*

42. More or less perversion of mental powers, attended with wakefulness and paroxysms of violence, with intervals of quiet; patient entirely unaware of his condition; eyes wild, and a greater or less wildness and fury marks the establishment of.............................. *Insanity.*

42. Digestion deranged; bowels constipated; fæces at first pale yellow, becoming grayish and at length white; at some time the conjunctiva and the whole skin becomes yellow; urine also colored with bile; there is also a most miserable, sick feeling.......................... *Jaundice.*

42. Entire loss of appetite; general tremors; incessant talkativeness; morbid wakefulness; suspicious, with great watchfulness; generally much unhealthy perspiration; at length the special senses become almost totally perverted, causing every kind of delusion, and the patient is in more or less constant alarm, generally without any desire to injure any one; his object is self-defense usually. *Delirium Tremens.*

42. Sudden or gradual; if gradual, attended with formication, pricking and the sensation of being asleep in the part; sometimes attended with imperfect or irregular spasms; and graduates into total loss of motion, with or without sensation; affected part wastes away, with a lowered temperature; the mind is also often more or less affected...................................... *Paralysis.*

42. Disease paroxysmal, with irregular intervals; sensation always perverted, resulting in strange, unusual and distressing sensations in different parts of the body; sometimes there is excessive laughter, crying, or other emotions; the disease may simulate any other nervous disorder, but can be known by its abrupt beginning and end, and the general history of the case; nutrition is unimpaired; pain common; cutaneous sensitiveness great; tenderness at some part of spine; if convulsions occur, there is no foaming at the mouth nor subsequent coma. *Hysteria.*

43. *High fever* an initial symptom of the disease........... 44
43. *High fever* not an initial symptom of the disease....... 47
44. Eruption a rash or a mere blush at first................ 45
44. Eruption pustular, vesicular, tubercular, bullar or squamous ........................................ 46
45. Coryza present, with cough; fauces exhibit a punctated redness. Eruption appears as small, distinct points like flea-bites, on the face first, disappearing under pressure; fever continues after eruption appears, which is in patches and rough; sometimes pimples appear among the rash; eruption generally occurs on fourth day............*Rubeola.*
45. Rash diffused, not perceptible to finger, appearing about *second* day, and decidedly punctate; sometimes containing small miliary vesicles; throat inflamed, fauces swollen, with stiffness of jaws; no cough present..........*Scarlatina.*
45. Eruptive rash beginning as a very limited spot and gradually spreads, hard and somewhat elevated, the marginal elevations distinct; redness disappears under pressure, but quickly returns, and is tender; blisters often appear; pain, swelling and tenderness of lymphatic glands of neck is an initial symptom..........................*Erysipelas.*
46. Disease *tubercular,* occurring as *buboes and carbuncles,* attended or preceded by darting pains in groin or armpit, the lymphatic glands swelling and suppurating......*Plague.*
46. Eruption *papular; fever subsiding on its appearance;* vomiting is often obstinate, and pain in back violent; the pimples afterwards become vesicular, then umbilicated, and finally pustular................................ *Variola.*
46. Eruption *vesicular,* often first appearing as small red spots, which soon become vesicular, with some itching and tingling; distinct and scattered eruption; fever subsides on its appearance................................ *Varicella.*
46. Disease caused by morbid inoculation, the seat of which first inflames; acute pains in the joints of the limbs, soon followed by small pustules, echymoses, blisters and tumors on different parts of the body; diarrhœa very constant; at length an offensive mucous, purulent or sanious discharge takes place from the nose...............*Glanders.*

47. Skin simply discolored, or covered with spots.......... 48
47. Disease clearly eruptive.............................. 49
48. Usually gradual; sensation of weariness; gums become swollen, rising up around the teeth, red, tender, and very disposed to bleed; face pale; breath offensive; feet and legs swollen and painful; and purplish spots or blotches appear upon the skin, generally first on the legs; generally occurs late in winter or early in spring..........*Scurvy.*
48. Sub or intra-cutaneous hemorrhage, generally commencing below the knee, making spots the color of which does not disappear under pressure, and unattended with either heat or itching; spots not elevated; blood blisters sometimes occur; gums sound; commonly a summer disease...... *Purpura Hæmorrhagica.*
48. Simple rose-colored splotches, more or less continuous, without itching, but sometimes with tingling, but not elevated; there is no itching if wheals occur; usually no constitutional symptoms.......................*Erythema.*
48. Constitutional symptoms subside on appearance of eruption, which occurs after two or three days, and consists of specks or small patches, which are sometimes distinct, sometimes confluent; usually appearing first on face and neck, and unattended with either cough, coryza or inflamed fauces....................................*Roseola.*
48. A disease of middle or advanced life; usually first appears on end of nose; the skin assumes a deep red color, followed by pustules; skin swells unequally; disease sometimes extends to face, and sometimes becomes tubercular; presents a very disgusting appearance.......*Rosacea.*
48. Erythematous patches, vividly red, with wheals rising among them, attended with *excessive itching;* eruption sometimes fugitive, coming on at particular hours, and of temporary duration...........................*Urticaria.*
49. Eruption papulous.................................. 50
49. Eruption vesicular................................. 51
49. Eruption pustulous................................. 52
49. Eruption squamous.................................. 53
49. Eruption tubercular................................ 54

49. Eruption bullar.............. .................. 55
50. A disease of infants, and consists in the appearance of numerous minute, florid *pimples*, with specks or patches of redness without elevation; pimples often attended with some pain and itching........................*Strophulus.*
50. Usually affecting adults; consists of small red *pimples* of a pin-head size, attended with heat, tingling and prickly itching; commonly a summer complaint, called "heat". *Lichen.*
50. The pimples of same color as sound skin, and attended with excessive itching; when broken by scratching, each pimple exhibits a small black scab of concrete exuded blood.......................................*Prurigo.*
51. Eruption *vesicular*, occurring in circumscribed patches, upon an inflamed base, which extends somewhat beyond their margins....................................*Herpes.*
51. Patches of very minute, closely crowded, glistening and transparent *vesicles*, with little or no intervening redness, with a disagreeable itching and tingling; the serum sometimes exudes and forms minute scales; the serum is sometimes very excoriating; vesicles rounded and flattened.......................................*Eczema.*
51. The first symptom is an itching, which arises from a minute accuminated *vesicle;* the vesicles are always distinct, and usually first appear between the fingers; pimples and pustules are sometimes mingled with them..........*Scabies.*
52. Eruption appears as distinct, conical, bright-red elevations, the size of split pea, often very painful; most frequently upon the neck and shoulders; they spread at base, and in three or four days become pustules, resembling small boils.............................*Echthyma.*
52. Eruption occurs in one or more clusters of minute yellow pustules, the size of a pin-head, on a circumscribed, red, somewhat elevated portion of skin; discharge somewhat profuse; scabs thick and abundant; eruption attended with very disagreeable heat, itching and smarting; does not cause the hair to fall off......................*Impetigo.*
52. Eruption occurs commonly on scalp as minute pustules, like yellow points, and appear to be set in the skin, and attended with itching; subjacent skin not inflamed; a pustule occurs at the root of each hair, the scab becomes cup-shaped; commonly called "scald-head," and is contagious.......................................*Porrigo.*

52. Chronic eruption of scattered and slow pustules, with inflamed and hardened bases, often terminating in tubercles; occurs on face and neck, never on legs; at first, small pimples appear; the skin between and around them *has an oily appearance;* pus small in quantity, quickly drying into a minute scab, which becomes a small, red, elevated spot, soon disappearing......................*Acne.*

52. Eruption consisting at first of minute pimples, which soon become pustular, occurring on *hairy parts of face,* particularly on upper lip and chin; each pustule is penetrated by a hair, is distinct, prominent, often pointed, and do not break until sixth or seventh day; then forming brownish, prominent scabs, intermingled with tubercles, sometimes as large as a cherry, which slowly suppurate.........................................*Sycosis.*

53. Appears in small papulous elevations the size of a pinhead, the summit of which have a slight scab; many occurring at once, being scaly; usually occurring on back and limbs; some itching attends them, especially when warm in bed; occur in irregularly circular patches, and are often attended with fissures and indentations....*Psoriasis.*

53. Eruption begins with minute solid eminences of a reddish color, which are quickly covered with delicate scales; patches very circular, with elevated reddish margins; the scales are tough, glistening, grayish or pale yellow, and translucent, accumulating and overlying each other around the margins of the patches; more apt to occur near joints.......................................*Lepra.*

53. Consists simply of patches of scales or scurf, minute, dry and whitish; often imbricated; sometimes occurs in erythematous spots or blotches, covered with scales, and sometimes look as if covered with meal or bran....*Ptyriasis.*

53. Commonly congenital, and occurs as a hardened, thickened, rough, almost horny state of cuticle, which breaks into small and irregular scale-like pieces, unattended with inflammation and having no definite outline........ *Ichthyosis*

54. Eruption consists of tumors from the size of a pea to that of a walnut, dusky red or livid at first, but at length bronze colored; intervening skin thickened and discolored; usually occurs first on face, when the eyebrows, lashes and beard come out; sensibility at length declines, and is lost; the tumors at length inflame, ulcerate and discharge an ichorous matter........ *Elephantiasis of Greeks.*

54. Eruption first appears as small red points like flea-bites, which soon rise into pimples as large as a pin-head, and then rapidly increase to a half or one inch in diameter; a fluid discharges which forms scabs, beneath which a fungous takes place; disease usually painless...... *Fambœsia.*

54. The lymphatic glands slowly enlarge, becoming hard and elastic, and if subcutaneous, more or less movable—under a sub-degree of inflammation; this at length increases, and the tumors suppurate, discharging pus or viscid serum, mixed with a curdy matter, and continuing indefinitely; the glands of the neck are first affected, then the groin, axillæ and breast......................... *Scrofula.*

55. Eruption consists of a succession of blisters, from the size of a pea to that of an egg, containing a *transparent* yellowish *fluid,* and end in forming THIN scabs; sometimes the bullæ are preceded by red circular spots of inflammation, at length becoming dusky................ *Pemphigus.*

55. Eruption consists of distinct *flattish bullæ, containing a serous, purulent or dark sanious fluid,* followed by *thick* scabs or ulcers; the surface on which they develop may or may not be inflamed.......................... *Rupia.*

---

A Remedy for Toothache.—A solution of gum copal in chloroform is a remedy for pain in decayed teeth. The cavity should be cleaned out, and a bit of cotton, moistened in the solution, introduced into it.

